# Electrocardiographic profile of adenosine pharmacological stress testing

**DOI:** 10.3892/etm.2015.2279

**Published:** 2015-02-09

**Authors:** HAO SUN, YUEQIN TIAN, LIHUI ZHENG, QINGRONG PAN, BOQIA XIE

**Affiliations:** 1 Department of Cardiology, Beijing Chaoyang Hospital, Capital Medical University, Beijing 100020, P.R. China; 2 Department of Nuclear Medicine, Cardiovascular Institute and Fuwai Hospital, Chinese Academy of Medical Sciences and Peking Union Medical College, Beijing 100037, P.R. China; 3 Department of Electrophysiology, Cardiovascular Institute and Fuwai Hospital, Chinese Academy of Medical Sciences and Peking Union Medical College, Beijing 100037, P.R. China; 4 Department of Endocrinology, Beijing Chaoyang Hospital, Capital Medical University, Beijing 100020, P.R. China

**Keywords:** adenosine, myocardial perfusion imaging, electrocardiography, arrhythmia, Chinese population

## Abstract

Adenosine stress testing in conjunction with radionuclide myocardial perfusion imaging has become a common approach for the detection of coronary artery diseases in patients who are unable to perform adequate levels of exercise. However, specific electrocardiographic alterations during the test have been rarely described. Using a Chinese population, the aim of the present study was to provide a detailed electrocardiographic profile of adenosine stress testing. The study population included 1,168 consecutive outpatients who had undergone adenosine-induced stress myocardial perfusion imaging. Electrocardiographic data during and immediately following the adenosine infusion were collected, and the corresponding myocardial perfusion images were assessed. During adenosine infusion, 174 transient and 47 persistent arrhythmic events occurred in 110 patients (9.42%). Furthermore, frequent premature atrial contractions occurred in 65 individuals and frequent premature ventricular contractions were observed in 73 individuals. Atrioventricular block (AVB) occurred in 75 patients [first degree (I°) AVB, 16; second degree (II°) AVB, 58; third degree AVB, 1), while sinoatrial block occurred in eight patients. ST depression emerged in 69 patients. Patients with a baseline I° AVB had an increased risk of a II° AVB, and patients exhibiting baseline ST depression were more likely to have a further depressed ST segment during the stress test (odds ratio, 28.68 and 5.01, respectively; both P<0.001). Following adenosine infusion, 10 patients (0.86%) exhibited newly occurred arrhythmic events. However, no patient presented with acute myocardial infarction or sudden mortality. In conclusion, the results demonstrated that adenosine infusion was a safe method, despite the relatively high incidence of arrhythmic events. The majority of arrhythmias that occurred during infusion were transient, were reversible with the termination of infusion and did not indicate abnormal perfusion results.

## Introduction

Pharmacological stress testing in conjunction with radionuclide myocardial perfusion imaging has been used as an alternative to dynamic exercise testing for the detection of coronary artery disease and risk stratification in patients who are unable to perform adequate levels of exercise (1–3). Adenosine is the most widely used agent due to its rapid onset of action and short half-life (<10 sec), which allows for dose titration. However, a number of side effects are frequently observed following the intravenous infusion of adenosine. In addition to flushing, nausea and dyspnea, arrhythmia is the most common side effect due to the negative chronotropic effect of adenosine (4–7). Although previous studies have demonstrated the overall safety of adenosine stress testing (8,9), specific electrocardiographic alterations during the process have been rarely described. In addition, whether the newly occurred arrhythmic events are within safe limits or whether they are indications of ischemia or/and are life-threatening is yet to be investigated. Moreover, whether adenosine infusion should be suspended upon the occurrence of severe arrhythmic events, including second degree (II°) and third degree (III°) atrioventricular block (AVB) or sinoatrial block (SAB), remains controversial. Shortage of the aforementioned information has impeded the wide application of adenosine stress testing since the method became available in 2003 in China (2,10). Therefore, the aim of the present study was to reveal the detailed characteristics of the electrocardiographic changes during an adenosine stress test, and to investigate the correlation between arrhythmia and perfusion results, in order to provide safety profiles of adenosine stress testing based on a Chinese population.

## Materials and methods

### Study population

Between May 2010 and January 2012, outpatients with potential diagnoses of coronary artery disease, who had undergone adenosine-induced stress using Technetium-99m sestamibi (99mTc-MIBI) single photon emission computed tomography (SPECT) myocardial perfusion imaging at Fuwai Hospital (Beijing, China), were prospectively enrolled in the study. The contraindications for adenosine stress testing included the occurrence of myocardial infarction within two months, unstable angina, hypotension (systolic blood pressure of <90 mmHg), hypertension (systolic or diastolic blood pressure of >200 or >110 mmHg, respectively), New York Heart Association (11)class IV congestive heart failure, an AVB greater than first degree (I°), patients with a pacemaker implantation or those with asthma or obstructive lung diseases. Ethical approval was obtained from the Ethics Review Board of Fuwai Hospital, and written informed consent was obtained from all the subjects enrolled.

### Adenosine infusion protocol

Adenosine (Shenyang Guangda Pharmaceuticals Co., Ltd., Shenyang, China) was infused at a constant rate of 140 μg/kg/min through a peripheral venous catheter, using an accurate computerized infusion pump (BYZ-810; Changsha BEYOND Medical Devices Co., Ltd., Changsha, China) over 6 min (total dose, 0.8 mg/kg body weight). At the third minute of adenosine infusion, 925 MBq 99mTc-MIBI (Radiation Chemistry Department, Beijing Normal University, Beijing, China) was injected as a bolus through the contralateral cubical vein and the adenosine infusion was continued for an additional 3 min. The heart rate and a 12-lead electrocardiogram (ECG) were recorded continuously at the baseline (at least 2 min prior to the infusion), during infusion and for at least 3 min after the termination of infusion. ECG data were analyzed by an experienced electrophysiologist, according to the 2008 AHA/ACCF/HRS recommendations for the standardization and interpretation of the ECG (12–14). The electrophysiologist was blinded to the myocardial perfusion results. Systolic and diastolic blood pressure were monitored every minute during the entire process. The administration of adenosine was terminated under the following circumstances: Patients with poorly tolerated side effects; severe hypotension (systolic blood pressure of <80 mmHg); horizontal or downsloping ST depression of >0.1 mV; ST elevation of >0.1 mV; crescendo II° or III° AVB or SAB.

### SPECT acquisition protocol

99mTc-MIBI SPECT myocardial perfusion imaging was performed 1.0–1.5 h after the completion of adenosine infusion using a dual-head gamma camera equipped with low-energy, high-resolution collimators (e.cam; Siemens Medical Solutions USA, Inc., Malvern, PA, USA). Projection data were acquired from 16 views over 180° from 45° right anterior oblique to 45° left anterior oblique, with 25 sec per view, on a 64×64 matrix. The image slices were analyzed visually by two experienced nuclear cardiologists in consensus based on 17 segments. Rest images were obtained the following day if the stress images were abnormal. The final perfusion results were determined by comparing the stressed images with the rest images. Reversible and irreversible defects were defined as ischemia and infarction, respectively, and if both patterns existed, the condition was defined as ischemia combined with infarction.

### Statistical analysis

Statistical analysis was performed using SPSS 19.0 software (IBM, Armonk, NY, USA). Continuous variables are expressed as the mean ± standard deviation, while categorical variables are presented as frequencies. The Student’s t-test was used to compare the differences in continuous variables, while the χ^2^ test was used to analyze the categorical variables. Logistic regression analysis was used to determine the risk factors. P<0.05 was considered to indicate a statistically significant difference.

## Results

### Patient characteristics

A total of 1,168 patients (male, 420; female, 748; mean age, 58±10 years) were enrolled in the study. Of these individuals, 330 patients had type 2 diabetes mellitus, 230 patients had hypertension and seven patients had undergone a previous percutaneous coronary intervention (Table I).

### Effects of adenosine infusion on hemodynamic parameters and cardiac electrical conduction

Blood pressure, heart rate and electrocardiographic intervals at the baseline, at the maximal response during adenosine infusion and at 2 min after the completion of adenosine administration are summarized in Table II. The intravenous adenosine infusion was demonstrated to induce a significant decrease in systolic blood pressure and an increase in the heart rate. In addition, adenosine infusion caused a prolongation of the PQ interval, without affecting the QRS interval. However, considering that an inverse ratio exists between an increasing heart rate and the shortening of the QT interval (15), the shortening of the QT interval may be caused by the increased heart rate rather than the infusion of adenosine. The maximal changes in the hemodynamic parameters and cardiac electrical conduction appeared between 2 and 3 min after the initiation of adenosine infusion. The parameters returned to the baseline level at 2 min after the termination of infusion.

### Baseline ECG characteristics

Baseline ECG characteristics are summarized in Table III. In total, 357 baseline arrhythmic events were observed in 340 patients (29.11%). A total of 73 patients (6.25%) exhibited sinus bradycardia (heart rate of <60 bpm), while 38 patients (3.25%) presented with sinus tachycardia (heart rate of >100 bpm). In addition, 74 patients (6.34%) had premature atrial contractions (>6 bpm) and 69 patients (5.91%) had premature ventricular contractions (>6 bpm). A I° AVB was identified in 22 patients (1.88%), and 41 patients (3.51%) exhibited atrial fibrillation. A total of 32 patients (2.74%) presented with a right bundle branch block, while eight patients (0.68%) exhibited a left bundle branch block. Furthermore, 96 patients (8.22%) exhibited baseline ST depression (>0.1 mV).

### ECG alterations during adenosine infusion

**Figure 1:**
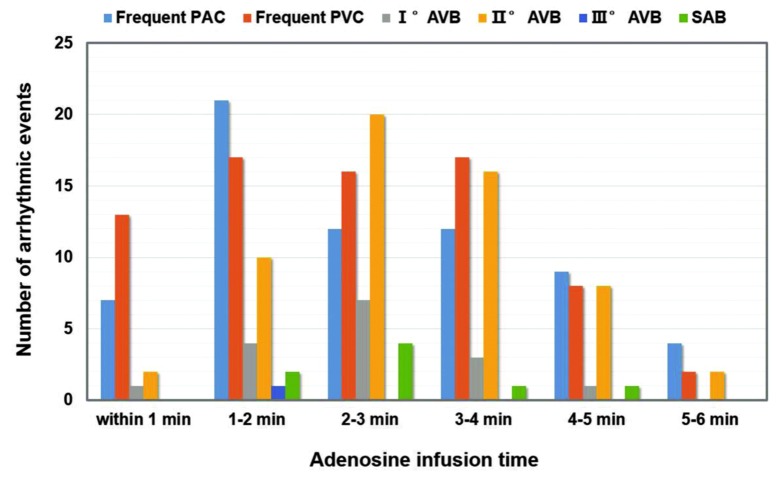
Emerging time of newly occurred arrhythmias during adenosine infusion. The majority of arrhythmic events emerged within the 2–3 min intervalafter adenosine infusion. PAC, premature atrial contractions; PVC, premature ventricular contractions; AVB, atrioventricular block; SAB, sinoatrial block; I°, first degree; II°, second degree; III°, third degree.

Newly occurred arrhythmias during adenosine infusion are summarized in Table IV and Fig. 1. During adenosine infusion, 221 arrhythmic events occurred in 110 patients (9.42%), among which 65 individuals (5.6%) had frequent premature atrial contractions and 73 patients (6.3%) exhibited frequent premature ventricular contractions. In total, 16 patients (1.4%) had I° AVB, 58 patients (5.0%) had II° AVB and one individual (0.09%) developed III° AVB following the development of II° AVB. In addition, eight individuals (0.68%) exhibited SAB. Of these arrhythmic events, 174 (14.90%) were transient (lasted for <10 sec), 34 (2.91%) were persistent (lasted for ≥10 sec) but self-terminated and 13 events (1.11%) were persistent and diminished following the termination of adenosine infusion. Early dose-termination was carried out in 15 patients. The newly occurred severe arrhythmias (SAB and II° or III° AVB) emerged when the infusion initiated and reached the maximal point during the 2–3 min interval following infusion (Fig. 1). The mean effective systolic blood pressure at the 2–3 min interval was 103±22 mmHg (baseline systolic blood pressure, 129±20 mmHg), which was considered to be a tolerable level.

Of the 1,168 patients, newly occurred ST depression (>0.1 mV) was observed in 69 patients (5.91%). During the adenosine stress test, no patient presented with acute myocardial infarction or sudden mortality, and no patient required specific treatment.

With regard to the correlations between the gender, age and baseline ECG characteristics of the patients and the development of II° AVB during adenosine infusion, only the baseline I° AVB was determined to be a predictor [P<0.001; odds ratio (OR), 28.68; 95% confidence interval (CI), 8.81–93.31; Table V). Furthermore, patients with a baseline ST depression were more likely to have a further depressed ST segment during the adenosine stress test (P<0.001; OR, 5.01; 95% CI, 2.76–9.10), possibly due to the already existing hypoperfusion prior to the test (Table VI).

### ECG alterations following the termination of adenosine infusion

Following the completion of adenosine infusion, 10 patients (0.86%) presented with newly occurred arrhythmias, including II° AVB in four patients, II° and III° AVB in one patient and SAB in five individuals (Table VII). The episodes were transient in nine patients; however, one patient had persistent SAB and ischemic ST changes due to a coronary spasm, which was revealed by an immediate coronary angiogram.

### Myocardial perfusion imaging results

Perfusion imaging revealed ischemia in 79 patients (6.76%), infarction in 10 patients (0.86%) and ischemia combined with infarction in seven patients (0.60%). Logistic regression analysis demonstrated that male patients and those who had newly occurred ST depression during adenosine infusion had an increased risk of abnormal perfusion results (OR, 2.14 and 95% CI, 1.35–3.4; OR, 14.66 and 95% CI, 8.12–26.48, respectively; both P<0.01; Table VIII).

## Discussion

In the present study, the detailed electrocardiographic changes through the entire process of the adenosine stress test were described. Adenosine was shown to have a strong depressant effect on the atrioventricular conduction system; however, an insignificant influence was observed on ventricular depolarization and repolarization. The newly occurred severe arrhythmias tended to emerge during the 2–3 min interval following adenosine administration, after which they gradually decreased. The majority of the newly occurred arrhythmias were transient and required no special treatment. In addition, no statistical correlation was observed between the newly occurred arrhythmias and abnormal perfusion results.

Adenosine is an autacoid that plays a critical role in regulating cardiac function. There are at least four subtypes of adenosine receptors, known as A1, A2A, A2B and A3, of which A2A is the predominant subtype responsible for coronary blood flow regulation (4). Documented studies have confirmed that adenosine-induced stress myocardial perfusion imaging has a relatively high sensitivity and specificity for the detection of coronary artery disease (1,2). Furthermore, this method offers a number of advantages when compared with the exercise test, including a rapid onset of action, a direct coronary vasodilatory effect, timely dose adjustment for its short half-life (<10 sec), a more standard operational procedure and a procedure that is less influenced by drugs (3). However, the unselected activation of adenosine receptors may lead to various undesirable side effects, among which ECG alterations are the most common due to the negative chronotropic effect of the A1 receptor, which suppresses the activity of the sinus node, atrioventricular junction and His-Purkinje system (4–7).

In the present study, the incidence of newly occurred AVB events was 6.42%, which is comparable with US population (7.63%) (8) and Japanese population (4.57%) (9) studies. Age was not found to be a predictor of the development of severe arrhythmia, indicating that adenosine may also be safe for elder Chinese patients. However, attention should be paid for patients with a baseline I° AVB, as these individuals were more likely to develop a II° AVB during adenosine infusion. Previous studies have demonstrated that new occurrence of ST depression during adenosine infusion is an independent predictor of future cardiac events (16–18). Consistently, in the present study, patients with newly developed ST depression were more likely to have abnormal perfusion results. Thus, attention should also be paid when ischemic ST changes emerge during adenosine infusion.

Finally, although the incidence is low (19–21), a coronary spasm may occur during or after the adenosine infusion. This may be due to the activation of the A1 receptor, which induces the contraction of vascular smooth muscle (22,23). In addition, delayed coronary spasms that occur at the termination of adenosine infusion may be the result of the withdrawal of vasodilatory effects and the reflected onset of vascular smooth muscle contraction (21). Therefore, intensive monitoring is highly recommended even following adenosine infusion.

The current preliminary study has a number of inherent limitations due to its single-centered research nature. In addition, the sample size was relatively small, which may lead to selection bias. Therefore, a randomized multicentered trial that includes a greater number of patients is required to confirm the ECG profiles of adenosine stress testing in a Chinese population.

In conclusion, based on a Chinese population, the findings of the present prospective study indicate the safety of adenosine pharmacological hyperemia in conjunction with radionuclide perfusion imaging. Despite the relative high incidence of arrhythmic events, the majority of arrhythmias that occurred during adenosine infusion were transient and did not indicate abnormal perfusion results.

## Figures and Tables

**Table I:** Patient characteristics.

Characteristics	Total (n=1,168)
Age (years)	58±10
Gender, male/female (n)	420/748
Weight (kg)	68±11
BMI (kg/m^2^)	25±3
Diabetes mellitus (n)	330
Hypertension (n)	230
Previous PCI (n)	7

BMI, body mass index; PCI, percutaneous coronary intervention.

**Table II:** Effects of the adenosine stress test on hemodynamic parameters and cardiac electrical conduction.

Parameter	Baseline	Peak effect	2 min after the termination of adenosine infusion
HR (bpm)	76±14	91±16^a^	83±15^b^
SBP (mmHg)	131±20	109±19^a^	115±19^a^
DBP (mmHg)	80±12	68±12^a^	74±12^b^
PQ interval (msec)	153±21	166±22^a^	154±20^b^
QRS interval (msec)	84±11	85±10	85±11^b^
QT interval (msec)	375±31	365±33^a^	373±31^b^

a P<0.05 and

b P>0.05, vs. baseline condition (paired t-test).

HR, heart rate; SBP, systolic blood pressure; DBP, diastolic blood pressure.

**Table III:** Baseline ECG characteristics.

Arrhythmia	Cases, n (%)
Sinus bradycardia	73 (6.25)
Sinus tachycardia	38 (3.25)
Frequent PAC	74 (6.34)
Frequent PVC	69 (5.91)
Atrial fibrillation	41 (3.51)
I° AVB	22 (1.88)
RBBB	32 (2.74)
LBBB	8 (0.68)
ST depression	96 (8.22)

ECG, electrocardiogram; PAC, premature atrial contractions; PVC, premature ventricular contractions; AVB, atrioventricular block; I°, first degree; RBBB, right bundle branch block; LBBB, left bundle branch block.

**Table IV:** Occurrence of new arrhythmic events during adenosine infusion.

		Transient arrhythmia^a^		Persistent arrhythmia^b^				
						
Arrhythmia	Total (n)	Cases (n)	Emerging time (sec)	Cases (n)	Emerging time (sec)	Duration (sec)	Self termination (n)	Early termination (n)
Frequent PAC	65	58	112±76	7	96±42	169±97	5	0
Frequent PVC	73	57	127±69	16	109±72	173±85	8	3
I°AVB	16	0	-	16	138±57	114±70	16	2
II° AVB	58	51	179±78	7	171±69	57±42	5	9
III° AVB	1	0	-	1	77	48	0	1
SAB	8	8	147±90	0	-	-	-	1
Total	221	174		47				15^c^

a Arrhythmia lasted <10 sec;

b Arrhythmia lasted >12 sec;

c One patient had a II° AVB that developed into III° AVB; thus, should not be double counted.

PAC, premature atrial contractions; PVC, premature ventricular contractions; AVB, atrioventricular block; I°, first degree; II°, second degree; III°, third degree; SAB, sinoatrial block.

**Table V:** Logistic regression analysis for the development of II° AVB during adenosine infusion.

Variables	OR	95% CI	P-value
Gender	1.09	0.61–1.95	0.77
Age	1.00	0.97–1.03	0.88
Baseline ST depression	0.00	0.00	1.00
Baseline sinus tachycardia	0.00	0.00	1.00
Baseline sinus bradycardia	0.38	0.10–1.46	0.16
Baseline I° AVB	28.68	8.81–93.31	0.001
Baseline RBBB	0.17	0.02–1.61	0.12
Baseline LBBB	0.45	0.03–6.16	0.55
Baseline PVC	0.57	0.13–2.41	0.44
Baseline PAC	0.53	0.12–2.29	0.40
Baseline atrial fibrillation	0.00	0.00	1.00

OR, odds ratio; CI, confidence interval; AVB, atrioventricular block; I°, first degree; RBBB, right bundle branch block; LBBB, left bundle branch block; PVC, premature ventricular contractions; PAC, premature atrial contractions.

**Table VI:** Logistic regression analysis for the development of ST depression during adenosine infusion.

Variables	OR	95% CI	P-value
Gender	0.95	0.55–1.62	0.84
Age	0.99	0.96–1.01	0.36
Baseline ST depression	5.01	2.76–9.10	0.001
Baseline sinus tachycardia	0.67	0.15–2.97	0.60
Baseline sinus bradycardia	0.76	0.26–2.27	0.63
Baseline I° AVB	0.00	0.00	1.00
Baseline RBBB	0.82	0.11–6.28	0.85
Baseline LBBB	0.00	0.00	1.00
Baseline PVC	0.92	0.32–2.71	0.88
Baseline PAC	1.14	0.42–3.05	0.80
Baseline atrial fibrillation	0.58	0.13–2.65	0.48
Newly occurred II° AVB	0.40	0.05–2.94	0.37
Newly occurred I° AVB	1.58	0.20–12.4	0.67
Persistent PVC	1.56	0.20–12.32	0.67
Persistent PAC	0.00	0.00	1.00
Newly occurred SAB	0.00	0.00	1.00

OR, odds ratio; CI, confidence interval; AVB, atrioventricular block; I°, first degree; II°, second degree; RBBB, right bundle branch block; LBBB, left bundle branch block; SAB, sinoatrial block; PVC, premature ventricular contractions; PAC, premature atrial contractions.

**Table VII:** Occurrence of arrhythmias following the termination of adenosine infusion.

Patient number	Gender	Age (years)	Baseline arrhythmia	Arrhythmia during infusion	Arrhythmia after infusion	Onset time^a^ (sec)	Arrhythmia duration (sec)	Treatment
79	F	41	None	None	SAB	311	12	PCI
248	F	50	Sinus bradycardia	II° AVB	SAB	27	9	None
672	F	59	None	None	II° AVB	49	3	None
762	F	62	None	None	SAB	145	2	None
773	F	62	Sinus bradycardia	None	SAB	120	2	None
851	F	65	None	None	II° AVB	12	8	None
925	M	67	None	None	II° AVB	12	2	None
984	F	69	None	None	II° AVB	38	3	None
1063	F	72	I° AVB	None	II°+III° AVB	64	7	None
1108	F	74	Sinus tachycardia	None	SAB	125	3	None

a Time following the initiation of infusion.

PCI, percutaneous coronary intervention; AVB, atrioventricular block; I°, first degree; II°, second degree; III°, third degree; SAB, sinoatrial block; F, female; M, male.

**Table VIII:** Logistic regression analysis for the occurrence of abnormal myocardial perfusion results.

Variables	OR	95% CI	P-value
Gender	2.14	1.35–3.40	0.001
Age	1.02	1.00–1.04	0.12
Baseline ST depression	0.74	0.33–1.63	0.45
Baseline sinus tachycardia	1.03	0.27–3.94	0.96
Baseline sinus bradycardia	1.36	0.57–3.22	0.49
Baseline I° AVB	1.03	0.20–5.22	0.97
Baseline RBBB	1.65	0.50–5.43	0.41
Baseline LBBB	0.00	0.00	1.00
Baseline PVC	1.00	0.39–2.56	1.00
Baseline PAC	1.12	0.47–2.69	0.80
Baseline atrial fibrillation	0.45	0.10–2.03	0.30
Newly occurred ST depression	14.66	8.12–26.48	0.001
Newly occurred II°AVB	1.00	0.33–3.05	1.00
Newly occurred I° AVB	0.83	0.09–7.33	0.86
Persistent PVC	0.00	0.00	1.00
Persistent PAC	0.00	0.00	1.00
Newly occurred SAB	0.00	0.00	1.00
Arrhythmia occurrence after adenosine infusion	1.96	0.24–16.36	0.53

OR, odds ratio; CI, confidence interval; AVB, atrioventricular block; I°, first degree; II°, second degree; RBBB, right bundle branch block; LBBB, left bundle branch block; SAB, sinoatrial block; PVC, premature ventricular contractions; PAC, premature atrial contractions.
